# Adsorption of Cadmium Ions from an Aqueous Solution on a Highly Stable Dopamine-Modified Magnetic Nano-Adsorbent

**DOI:** 10.1186/s11671-019-3154-0

**Published:** 2019-11-28

**Authors:** Ting Lei, Sheng-Jian Li, Fang Jiang, Zi-Xuan Ren, Li-Lian Wang, Xiang-Jun Yang, Li-Hong Tang, Shi-Xiong Wang

**Affiliations:** Key Laboratory of Medicinal Chemistry for Natural Resource (Yunnan University), Ministry of Education, School of Chemical Science and Technology, Yunnan University, No. 2, Cuihu North Road, Kunming, 650091 China

**Keywords:** Magnetic nanomaterials, Dopamine modification, Adsorption, Cadmium

## Abstract

**Abstract:**

Magnetic nanomaterials were functionalized with dopamine hydrochloride as the functional reagent to afford a core–shell-type Fe_3_O_4_ modified with polydopamine (Fe_3_O_4_@PDA) composite, which was used for the adsorption of cadmium ions from an aqueous solution. In addition, the effects of environmental factors on the adsorption capacity were investigated. Furthermore, the adsorption kinetics, isotherm, and thermodynamics of the adsorbents were discussed. Results revealed that the adsorption of cadmium by Fe_3_O_4_@PDA reaches equilibrium within 120 min, and kinetic fitting data are consistent with the pseudo-second-order kinetics (*R*^2^ > 0.999). The adsorption isotherm of Cd^2+^ on Fe_3_O_4_@PDA was in agreement with the Freundlich model, with the maximum adsorption capacity of 21.58 mg/g. The thermodynamic parameters revealed that adsorption is inherently endothermic and spontaneous. Results obtained from the adsorption–desorption cycles revealed that Fe_3_O_4_@PDA exhibits ultra-high adsorption stability and reusability. Furthermore, the adsorbents were easily separated from water under an enhanced external magnetic field after adsorption due to the introduction of an iron-based core. Hence, this study demonstrates a promising magnetic nano-adsorbent for the effective removal of cadmium from cadmium-containing wastewater.

**Graphical Abstract:**

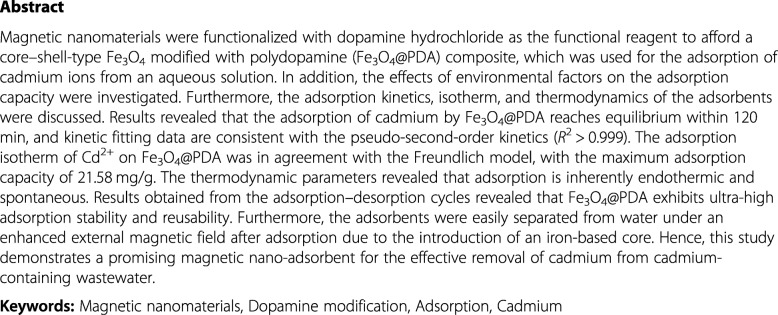

## Introduction

Pollution by Cd(II) metals has become one of the serious environmental problems. A majority of the cadmium pollution originates from the smelting of non-ferrous metals, sintering of ores, discharge of wastewater from the electroplating industry, and preparation of phosphate fertilizers from phosphate rock [1–4]. With a long half-life, cadmium is slowly metabolized in the human body; hence, it can be easily accumulated in organs such as the kidneys of the human body [5]. The long-term exposure of humans or animals to low cadmium concentrations can lead to health issues, including kidney dysfunction and reproductive organ and bone damage, as well as malformation of the development of offspring [6]. Hence, Cd(II) is designated as a carcinogen by the World Health Organization, the International Agency for Research on Cancer, and the National Toxicology Program (USA) [7–9]. Therefore, an effective removal method of cadmium ions for the purpose of reducing environmental pollution and damage to humans and animals is an interesting topic for environmental governance.

Currently, cadmium can be removed by chemical precipitation, ion exchange, adsorption, solvent extraction, and membrane separation [10–14]. In particular, adsorption has been widely employed due to its simple operation, high efficiency, and cost-effectiveness [15]. In the past two decades, various adsorbent materials have been developed and utilized, including natural soil materials, inorganic minerals, activated carbon, zeolites, silica gel, chitosan, and polymer materials [16–22]. Compared to these adsorbents, magnetic nano-adsorbents can be developed and used for treating industrial wastewater due to their high specific surface area, good biocompatibility, cost-effectiveness, and rapid separation and recovery under an external magnetic field [23]. However, the superparamagnetism and high surface energy of magnetic Fe_3_O_4_ lead to facile agglomeration or corrosion and poor stability [24, 25]. Hence, Fe_3_O_4_ needs to be functionalized for the improvement of its dispersibility, stability, and contaminant removal rate. Currently, the main surface modifiers of magnetic nanoparticles include organic small molecules, high molecular-weight polymers, inorganic materials, and metal–organic frameworks [26–29].

In the past decade, dopamine (DA) has been reported to form a stable polydopamine (PDA) film with controlled thickness by self-polymerization under weakly alkaline conditions. Studies have revealed that PDA is a highly adhesive biopolymer with functional groups such as catechol, amine, and imine, which can adhere to the surface of organic or inorganic materials via the formation of covalent and non-covalent interactions (e.g., chelation, hydrogen bonds, van der Waals forces, and π–π stacking) [30, 31]. In addition, these interactions between PDA and the carrier exist between PDA and water pollutants, thereby providing a method for removing water-containing contaminants. Farnad et al. [32] have synthesized PDA nanoparticles with an average diameter of 75 nm that can efficiently adsorb Cu^2+^ from wastewater. The maximum adsorption capacity of 34.4 mg/g is obtained after the reaction is conducted for 270 min at pH 5. Zhang et al. [33] have obtained PDA-modified magnetic nanoparticles (Fe_3_O_4_/PDA) and subsequently used them for the removal of methylene blue, lemon yellow, Cu^2+^, Ag^+^, and Hg^2+^ from sewage. At an optimum pH, the maximum adsorption capacities of Fe_3_O_4_/PDA for these contaminants are 204.1, 100.0, 112.9, 259.1, and 467.3 mg/g, respectively. This study demonstrated the immense potential of Fe_3_O_4_/PDA for the removal of multiple pollutants. Huang et al. [34] have prepared a PDA-coated clay (D-clay/Fe^3+^) with a three-dimensional network structure by using Fe^3+^ for coordination with PDA. As-prepared material exhibits good elastic response and self-repairing ability. Rhodamine 6G (Rh6G) can be removed from water through the π–π stacking interactions of the aromatic moiety between PDA and Rh6G. Gao et al. [35] have prepared a PDA-modified graphene hydrogel (PDA-GH) by a one-step approach. This material exhibits high adsorption capacities for Pb^2+^, Cd^2+^, rhodamine B, and p-nitrophenol. This material is also easily regenerated using low-cost desorbents such as acids and alcohols. Hence, according to previous studies, PDA exhibits a good adsorption capacity for various pollutants (i.e., heavy metals and organic pollutants), and it demonstrates broad application prospects for wastewater treatment.

**Scheme 1:**
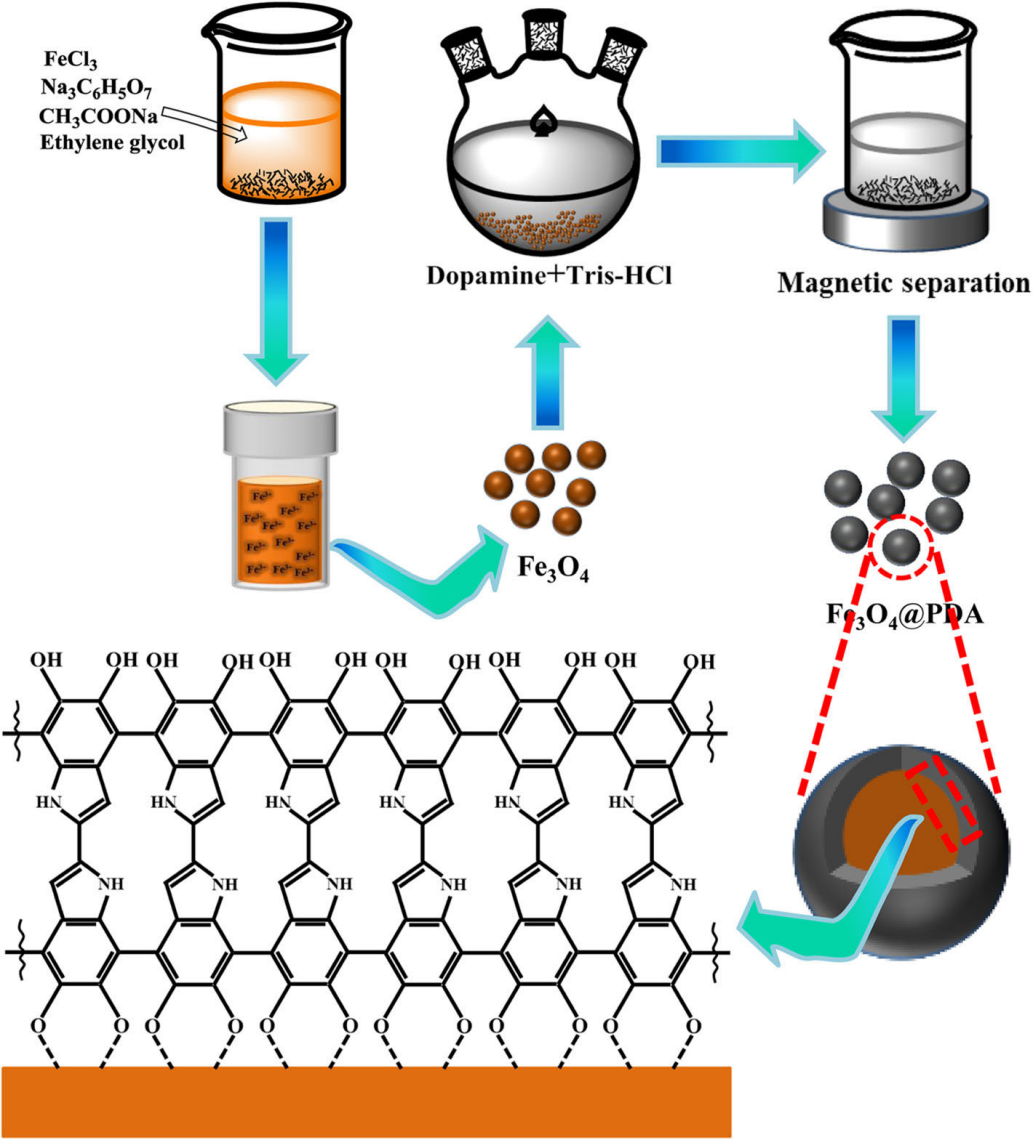
Synthesis and structure of Fe_3_O_4_@PDA

Herein, Fe_3_O_4_ was synthesized by a solvothermal method and coated with PDA as the modifier. The amino and phenolic hydroxyl groups on PDA were utilized for the adsorption of cadmium ions, which aimed at exploring the feasibility of using Fe_3_O_4_ modified with polydopamine (Fe_3_O_4_@PDA) as the adsorbent to remove cadmium ions for wastewater treatment. In addition, the effects of various environmental parameters on the adsorption performance of Cd^2+^ on Fe_3_O_4_@PDA microspheres were systematically investigated by batch adsorption experiments. Furthermore, the kinetics, adsorption isotherms, thermodynamics, reusability, and stability of Fe_3_O_4_@PDA were evaluated. As-synthesized Fe_3_O_4_@PDA exhibited advantages of good stability, low biological toxicity, mild synthesis conditions, facile separation and recovery, environmental friendliness, and lack of secondary pollution. Scheme 1 shows the synthesis and structure of Fe_3_O_4_@PDA.

## Methods

### Materials

Sodium acetate anhydrous, ethylene glycol, and nitric acid (65–68 wt%) were purchased from Guangdong Chemical Reagent Engineering-Technological Research and Development Center (Guangdong, China). Trisodium citrate dihydrate and hydrochloric acid (36–38 wt%) were purchased from Tianjin Fengchuan Chemical Reagent Technologies Co., Ltd. (Tianjin, China). Sodium hydroxide, ferric chloride hexahydrate, and cadmium chloride hydrate (99%) were obtained from Guangfu Technology Development Co., Ltd. (Tianjin, China). Tris (hydroxymethyl) aminomethane and 3-hydroxytyramine hydrochloride were purchased from Aikeda Chemical Reagent Co., Ltd. (Chengdu, China). All chemicals were of analytical grade or better, and they were used without further purification. Ultrapure water was used throughout the experimental process.

### Synthesis of Fe_3_O_4_ Microspheres

Fe_3_O_4_ magnetic nanoparticles were prepared by a solvothermal reaction. Briefly, FeCl_3_·6H_2_O (8.1 g), Na_3_C_6_H_5_O_7_·2H_2_O (6 g), and CH_3_COONa (21.6 g) were first dissolved in ethylene glycol (240 mL) with magnetic stirring. Second, after vigorous magnetic stirring for 30 min, the homogeneous orange-red solution was divided into three parts (80 mL/part) and transferred into three Teflon-lined stainless-steel autoclaves (100 mL) and sealed for heating at 200 °C. After reaction for 8 h, the autoclave was cooled to room temperature. The obtained Fe_3_O_4_ particles were collected using an external magnet and washed several times with ethanol and H_2_O. Finally, the products were stored in an appropriate amount of ethanol for further use.

### Synthesis of Fe_3_O_4_@PDA Particles with Core–Shell Nanostructures

PDA-coated Fe_3_O_4_ nanoparticles were obtained by the polymerization of DA in an alkaline buffer at 25 °C. Briefly, the synthesized Fe_3_O_4_ particles were added into a 1000-mL three-necked flask containing 200 mL of Tris-HCl buffer (10 mM, pH 8.5) and subjected to sonication for 5 min. Then, 2 g of dopamine hydrochloride was weighed and dispersed in a 500-mL beaker containing 400 mL of Tris-HCl buffer (10 mM, pH 8.5) and subjected to sonication for 1 min. Then, it was added into the three-necked flask and mechanically stirred for 24 h. The synthesized Fe_3_O_4_@PDA particles were separated and collected using a magnet and washed several times with deionized water and ethanol, followed by drying under vacuum at 50 °C for 4 h.

### Characterization

X-ray diffraction (XRD) was employed to identify the crystalline structure and phase composition of the synthesized samples in the 2*θ* range from 10 to 90° using Co Kα radiation. The morphology and dimensions of the samples were observed by transmission electron microscopy (TEM). Fourier transform infrared (FTIR) spectra of the samples were recorded in the wavenumber range of 400–4000 cm^−1^. The chemical states of the samples were examined by X-ray photoelectron spectroscopy (XPS). The specific surface area was measured by *N*_2_ adsorption using the Brunauer–Emmett–Teller (BET) method. The magnetization curve was measured at room temperature under a varying magnetic field from − 20,000 to 20,000 Oe using an MPMS-3 vibrating sample magnetometer (VSM).

### Batch Adsorption Studies

Adsorption properties of the two adsorbents (i.e., Fe_3_O_4_ and Fe_3_O_4_@PDA, respectively) for the cadmium ions from an aqueous solution under various operating conditions were investigated. Adsorption experiments were carried out in an Erlenmeyer flask with 20.0 mg of the adsorbent and 50 mL of a 20 mg/L Cd^2+^ solution (pH 7). The Erlenmeyer flask was placed in a constant-temperature shaker and subjected to shaking at 250 rpm for 120 min at 25 °C. After attaining equilibrium, the magnetic nano-adsorbent was separated from the Cd^2+^ solution by using an external magnetic field. Then, the supernatant was removed, and the concentration of cadmium ions in the initial and adsorbed solutions was estimated by atomic absorption spectrometry.

To investigate the effect of the reaction time, the reaction time was set between 15 and 2160 min. The effect of the initial cadmium solution concentration on the adsorption capacity of the adsorbent was investigated by the variation in the initial Cd^2+^ concentration between 3 and 30 mg/L. In addition, the effect of the adsorbent dose was investigated by the variation in the adsorbent dose (10–50 mg). The effect of pH on adsorption was investigated by the addition of 0.1 M NaOH or 0.1 M HCl to adjust the solution pH in the range of 4.0–9.0. Experiments were carried out at 20–45 °C to examine the effect of temperature on the adsorption performance.

The adsorption capacity for cadmium was calculated as follows:

1 $$ {q}_e=\left({C}_0-{C}_e\right)\frac{V}{M} $$

where *C*_0_ (mg/L) is the initial metal ion concentration, *C*_*e*_ (mg/L) is the equilibrium solution concentration, *V* (L) is the total solution volume, and *M* (g) is the mass of magnetic nanoparticles.

### Reusability and Stability Studies

The reusability and stability of the adsorbent were investigated by performing 10 adsorption–desorption cycles using the Fe_3_O_4_@PDA adsorbent. The adsorption capacity of the adsorbent for Cd^2+^ in each cycle was analyzed. Experimental conditions for the adsorption reaction were as follows: 20 mg Fe_3_O_4_@PDA, 50 mL of a 20 mg/L CdCl_2_ solution (pH 7), and reaction at 250 rpm for 120 min at 25 °C. Desorption was carried out using 50 mL of 0.5 M HCl as the desorbent, and the reaction was carried out at 250 rpm for 60 min at 25 °C. The adsorbent was separated by using a magnet after desorption and washed with deionized water and ethanol until neutral pH was achieved. After drying the adsorbents at 60 °C for 30 min, the next adsorption–desorption experiment was carried out.

## Results and Discussion

### Characterization of the Magnetic Adsorbents

**Fig. 1:**
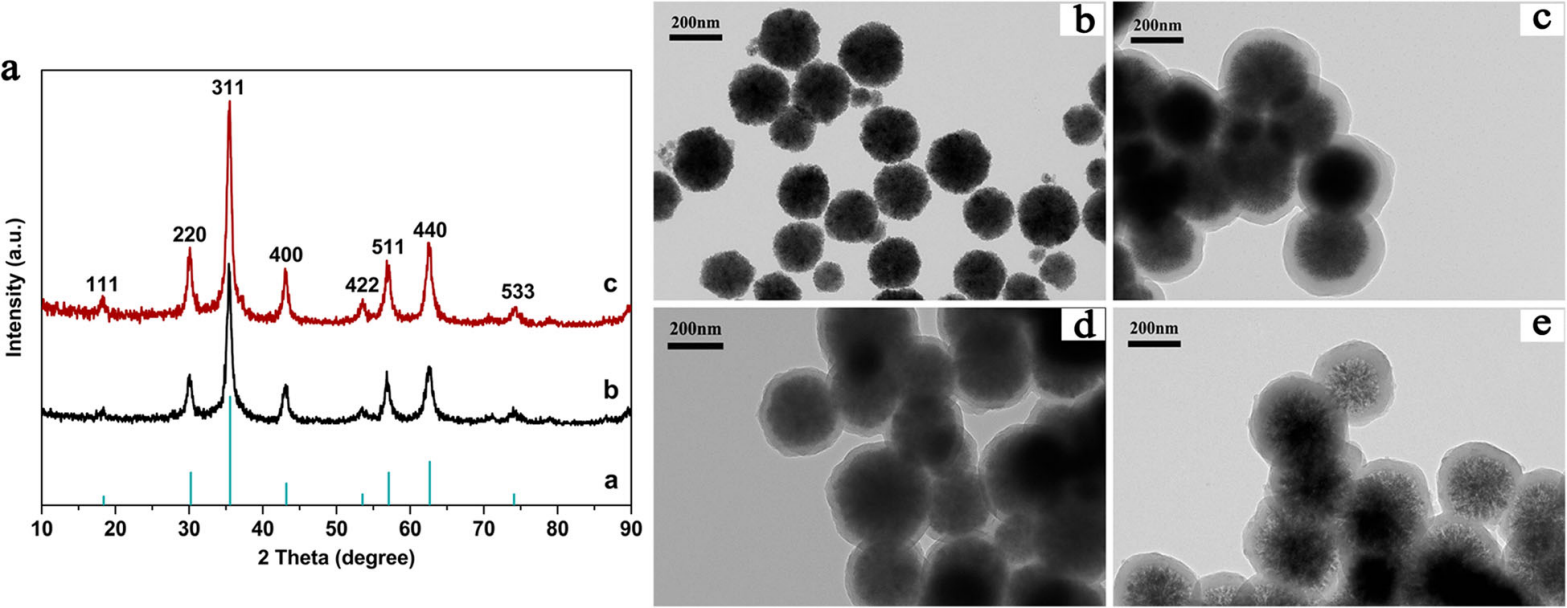
**a** X-ray diffraction (XRD) patterns of (a) standard Fe_3_O_4_, (b) synthesized Fe_3_O_4_, and (c) Fe_3_O_4_@PDA; TEM images of **b** Fe_3_O_4_, **c** Fe_3_O_4_@PDA, **d** Fe_3_O_4_@PDA-Cd^2+^, and **e** Fe_3_O_4_@PDA of the 10th adsorption–desorption cycles

Figure 1a shows the XRD patterns of the adsorbents. The observed diffraction peaks of Fe_3_O_4_ (Fig. 1a—(b)) were consistent with the standard face-centered cubic (fcc) Fe_3_O_4_ (JCPDS card number 19-629) (Fig. 1a—(a)) [36], and impurity peaks were not observed. Relatively strong diffraction peaks were observed at 2*θ* values of 18°, 30°, 35.4°, 43°, 53°, 56°, 62°, and 73°, corresponding to the standard diffraction peaks of the (111), (220), (311), (400), (422), (511), (440), and (533) crystallographic planes of Fe_3_O_4_, respectively, indicating that the synthesized magnetic nanoparticles are Fe_3_O_4_ and not other ferrites. The Fe_3_O_4_ particles synthesized herein exhibited cluster-like nanostructures, comprising several secondary Fe_3_O_4_ nanoparticles. The average size of the secondary nanocrystals at the (311) crystal plane observed in the XRD patterns was estimated to be ~ 16 nm by the Debye–Scherrer equation [37]. In the XRD pattern of Fe_3_O_4_@PDA particles (Fig. 1a—(c)), the main peaks were similar to those observed for original Fe_3_O_4_, and as the PDA shell was amorphous, diffraction peaks corresponding to PDA were not observed. The result revealed that the Fe_3_O_4_ crystal structure is well retained after coating with PDA. Extremely sharp diffraction peaks corresponding to Fe_3_O_4_ and Fe_3_O_4_@PDA were observed, indicative of the better crystallinity for as-prepared Fe_3_O_4_ [38].

Furthermore, TEM images were recorded to observe the morphology of the adsorbent and to confirm the polymerization of DA on the Fe_3_O_4_ core. Figure 1b shows the TEM image of bare Fe_3_O_4_ particles. As can be clearly observed in the TEM image, as-synthesized Fe_3_O_4_ exhibited a cluster of nanospheres comprising several secondary Fe_3_O_4_ nanoparticles. With the addition of sodium citrate as the stabilizer during the synthesis, the as-obtained Fe_3_O_4_ particles exhibited a uniform size (average particle diameter of 250–300 nm) and good dispersibility without agglomeration. The Fe_3_O_4_@PDA composites exhibited a core–shell structure (Fig. 1c). As the core, dark Fe_3_O_4_ particles were uniformly coated by light-colored PDA, with a 40–50-nm-thick PDA layer. Furthermore, the Fe_3_O_4_@PDA nanoparticles tended to agglomerate, likely related to the magnetic attraction [25] and hydrogen bonding between the Fe_3_O_4_@PDA particles. In addition, owing to the high surface energy of the nanoparticles, the system automatically changed in the direction of the decreased surface area, leading to the nanoparticle agglomeration [39]. Figure 1d shows the Fe_3_O_4_@PDA particles after the adsorption of cadmium ions. The PDA coating was darker in color, and the core structure was unchanged. Moreover, the dispersibility of Fe_3_O_4_@PDA-Cd^2+^ was better than that of Fe_3_O_4_@PDA particles due to the decrease in the surface energy of Fe_3_O_4_@PDA after the adsorption of Cd^2+^. By the comparison of Fig. 1d and e, after 10 adsorption–desorption cycles, the PDA coating remained intact, but the Fe_3_O_4_ core density decreased because the desorbent (0.5 mol/L HCl) corroded a part of the secondary Fe_3_O_4_ nanoparticles. Although the core was corroded, the Fe_3_O_4_@PDA structure was retained after 10 adsorption–desorption cycles, indicating that PDA exhibits a good protective effect on the exposed Fe_3_O_4_ particles.

The BET specific surface areas of Fe_3_O_4_ and Fe_3_O_4_@PDA were estimated to be 61.84 m^2^/g and 14.23 m^2^/g, respectively. The decrease in the specific surface area of Fe_3_O_4_@PDA corresponded to the increase in the particle size of the magnetic nanoparticles after coating with DA. In addition, the agglomeration of Fe_3_O_4_@PDA led to the decrease in the specific surface area [40].

**Fig. 2:**
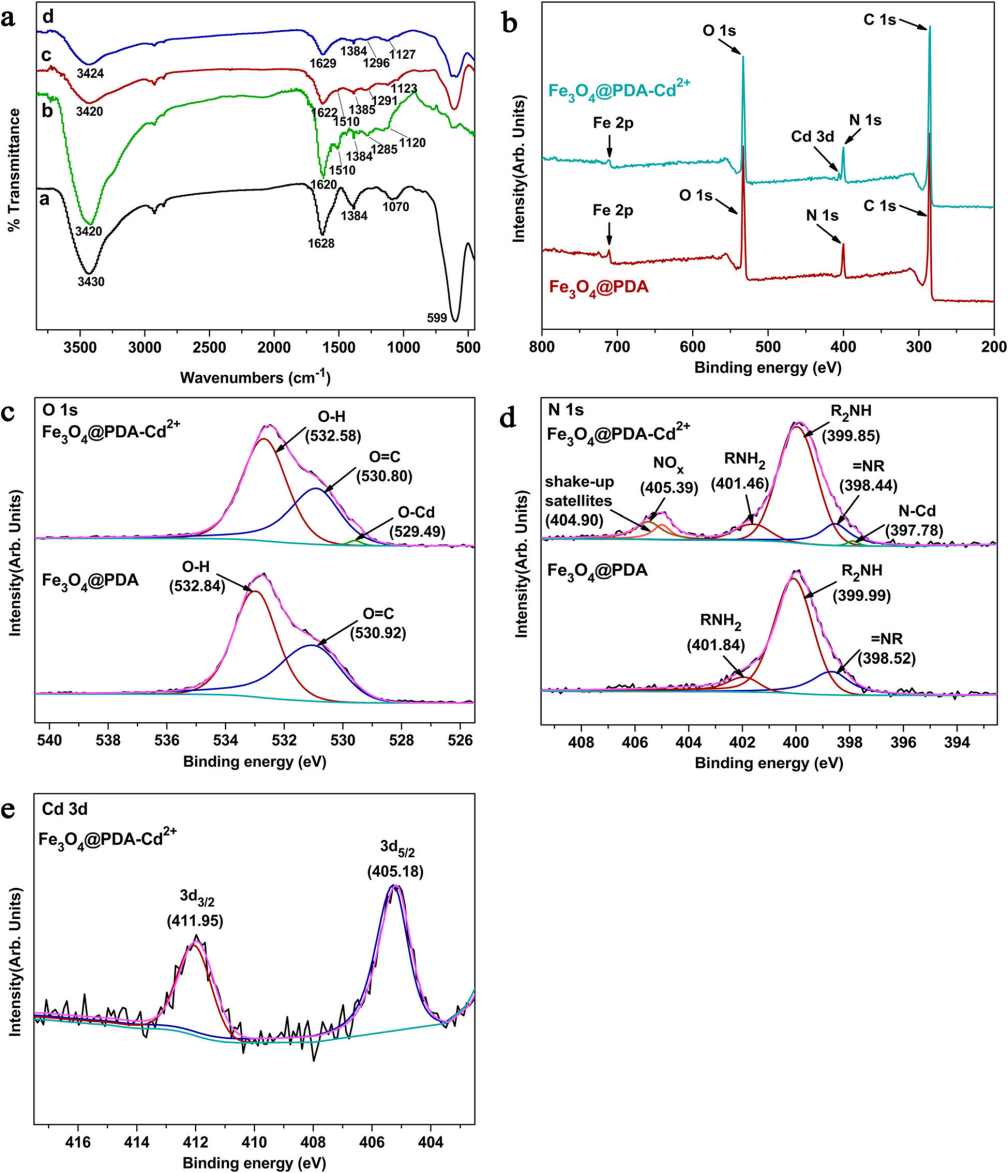
**a** FTIR spectra of (a) Fe_3_O_4_, (b) PDA microspheres, (c) Fe_3_O_4_@PDA, and (d) Fe_3_O_4_@PDA-Cd^2+^; XPS spectra of **b** wide-scan, **c** O 1s, **d** N 1s, and **e** Cd 3d

Figure 2a shows the FTIR spectra of Fe_3_O_4_, PDA, Fe_3_O_4_@PDA, and Fe_3_O_4_@PDA-Cd^2+^. A strong absorption peak observed 599 cm^−1^ corresponded to the Fe–O–Fe bond (Fig. 2a—(a)) [41]. In this study, sodium citrate was added to improve the dispersibility of Fe_3_O_4_; hence, absorption peaks observed at 1628 and 1384 cm^−1^ in the FTIR spectrum of Fe_3_O_4_ correspond to the residual carboxylate group [42]. In addition, the bands observed at 1070 and 3430 cm^−1^ corresponded to the C–H bending vibration and stretching vibrations for surface-adsorbed O–H of water, respectively [43]. In the FTIR spectrum of PDA microspheres (Fig. 2a—(b)), the peak observed at 1620 cm^−1^ corresponded to the stretching vibration of the aromatic ring and the bending vibration of N–H [44]. The peak observed at 1510 cm^−1^ corresponded to the N–H shearing vibration of the amino group, and the peaks observed at 1384 and 1285 cm^−1^ corresponded to the bending and stretching vibrations of C–O–H, respectively, and the peak observed at 1120 cm^−1^ corresponded to the C–O stretching vibrations [45]. The peak corresponding to PDA at 3430 cm^−1^ was broader than that observed in the FTIR spectrum of Fe_3_O_4_, possibly related to the superposition of the stretching vibrations of the amino group, phenolic hydroxyl group, and adsorbed water in the DA polymer [40]. Absorption peak characteristics of Fe_3_O_4_ and PDA polymers in the FTIR spectrum of the Fe_3_O_4_@PDA composites were observed [Fig. 2a—(c)]. Therefore, the results obtained from FTIR spectroscopy revealed that DA is successfully coated on the Fe_3_O_4_ surface and that the Fe–O–Fe peak of Fe_3_O_4_@PDA is weaker than that of Fe_3_O_4_, indicating that the thicker PDA coating exhibits a certain shielding effect. In the FTIR spectrum of Fe_3_O_4_@PDA-Cd^2+^ (Fig. 2a—(d)), after the adsorption of heavy metal ions, Fe_3_O_4_@PDA exhibited a peak shift from 3 to 7 for the peaks observed at 3420, 1622, 1291, and 1123 cm^−1^, with a slightly weakened peak intensity. All of these peaks corresponded to amino and phenolic hydroxyl groups, indicating that these two groups are the main functional groups that interact with metal ions.

**Scheme 2:**
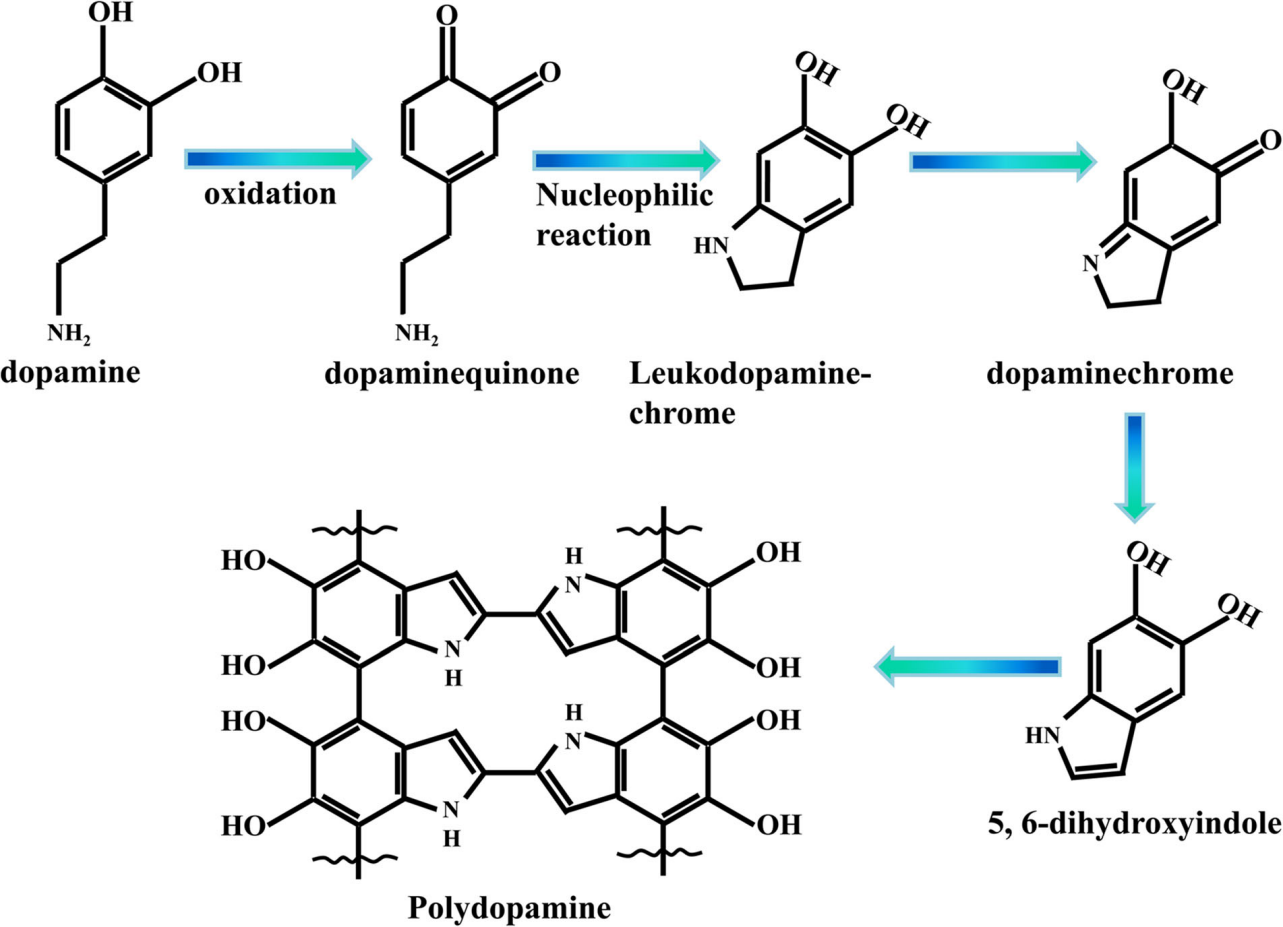
Synthetic of polydopamine

To further verify the FTIR spectra, XPS profiles of Fe_3_O_4_@PDA before and after adsorption were recorded. The wide-scan spectrum revealed C 1s (285.90 eV), N 1s (400.28 eV), O 1s (532.72 eV), and Fe 2p (711.22 eV) peak characteristics of Fe_3_O_4_@PDA, and the peak intensity revealed that the surface oxygen content of Fe_3_O_4_@PDA is greater than that of nitrogen (Fig. 2b). The Cd 3d peak was observed in the wide-scan spectrum of Fe_3_O_4_@PDA-Cd^2+^, indicating that Cd^2+^ can be adsorbed on the Fe_3_O_4_@PDA surface. To verify the peaks observed in the FTIR spectra, Cd^2+^ interacted with -OH and -NH_2_ on the adsorbent surface. Figure 2c and d show the comparison of the O 1s and N 1s binding energies for Fe_3_O_4_@PDA before and after adsorption, respectively. Binding energy peaks in the O 1s spectrum before adsorption were observed at 532.58 and 530.80 eV, corresponding to O in -OH and -C=O [46], respectively. Among these peaks, -C=O was obtained by the conversion of the phenolic hydroxyl groups into quinone (Scheme 2). By the comparison of the peak areas of -OH and -C=O, the -OH content on the adsorbent surface is known to be greater than the -C=O content. After the adsorption of cadmium, the binding energies of -OH and -C=O shifted to low binding energies due to the donation of the lone-pair electrons of O to Cd^2+^, leading to the decrease in the electron cloud density of O [47]. The new peak observed at 529.49 eV was speculated to be related to the complexation of Cd^2+^ with O on the Fe_3_O_4_@PDA surface. In the N 1s spectrum before adsorption, =N–R (398.52 eV), R_2_N–H (399.99 eV), and R–NH_2_ (401.84 eV) were observed [48]. The three peaks observed in the adsorbed N 1s spectrum shifted toward the direction of low binding energy, corresponding to the decrease in the electron cloud density of N caused by the sharing of the lone-pair electrons of N with Cd^2+^. Based on this result, the new peak observed at 397.78 eV was presumed to originate owing to the complexation of Cd^2+^ with N. As the purity of the used reagent (CdCl_2_) was 99%, N-containing impurities were introduced during the adsorption; hence, peaks observed at 404.90 and 405.39 eV correspond to the shake-up satellites and nitrogen oxides, respectively [49]. The peak area and peak intensity of O–Cd and N–Cd were low, indicating that only a part of O and N on Fe_3_O_4_@PDA participates in the reaction. The peaks of Cd 3d (Fig. 2e) were divided into the main peak of Cd 3d_5/2_ (405.18 eV) and the secondary peak of Cd 3d_3/2_ (411.95 eV). Generally, the binding energy of Cd 3d_5/2_ of CdO is 404.8 eV [50]; however, the binding energy of Cd 3d_5/2_ in this study was 405.18 eV, indicating that Cd^2+^ interacted with not only oxygen functional groups but also nitrogen functional groups.

Hence, the O 1s, N 1s, and Cd 3d XPS spectra confirmed the results obtained from FTIR spectroscopy; Cd^2+^ is adsorbed by the action of the amino and hydroxyl groups on the Fe_3_O_4_@PDA surface; and the hydroxyl group plays a major role.

**Fig. 3:**
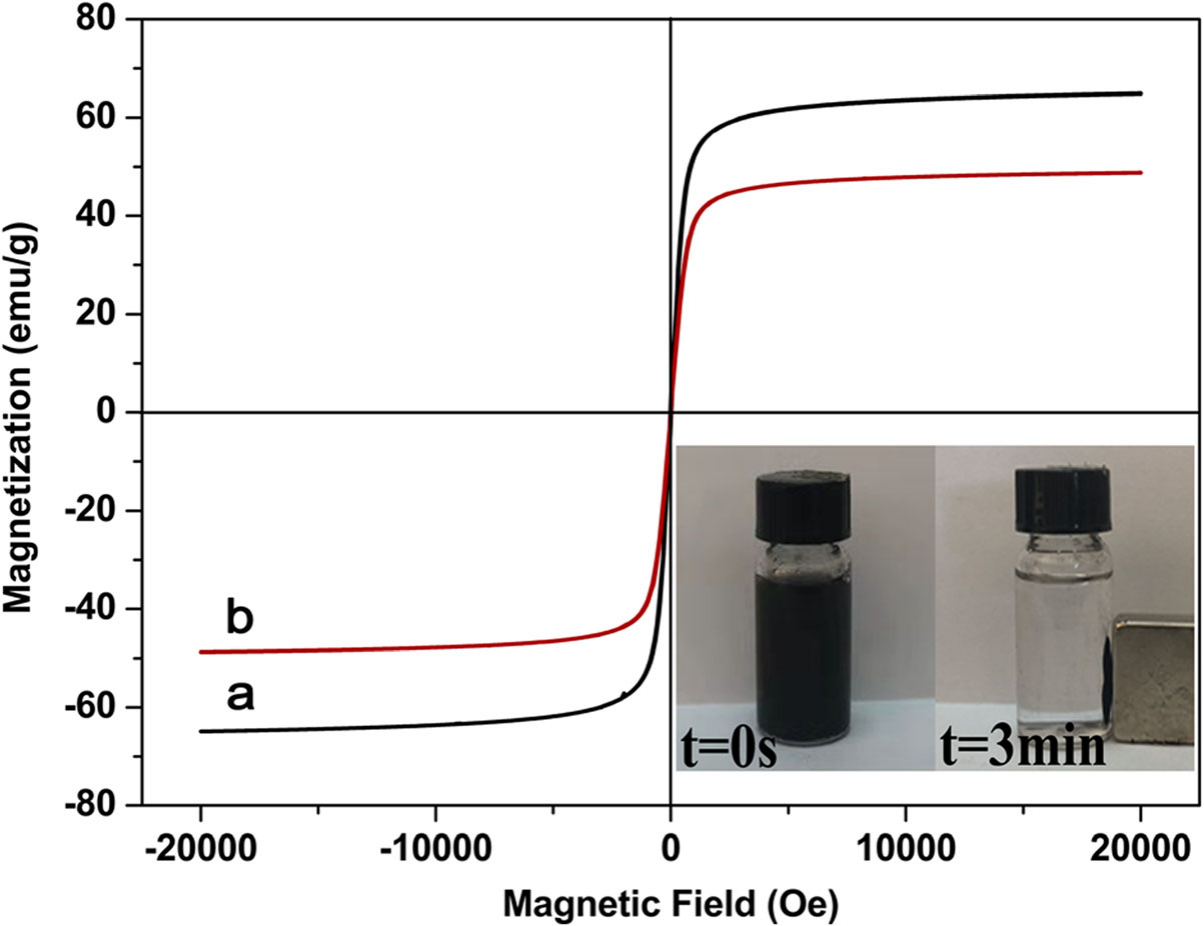
Magnetization curves of **a** Fe_3_O_4_ and **b** Fe_3_O_4_@PDA nanoparticles at room temperature

To investigate the magnetic properties of the materials, the VSM was utilized to measure the hysteresis curve of the materials. With the increase in the magnetic field strength, the magnetization of Fe_3_O_4_ and Fe_3_O_4_@PDA increased (Fig. 3). The saturation magnetization value of Fe_3_O_4_ nanoparticles was estimated to be 64.9 emu/g, while that of the Fe_3_O_4_@PDA composite was reduced to 48.8 emu/g. This decrease was related to the deposition of a large number of non-magnetic DA polymers on Fe_3_O_4_. Hysteresis was not observed in the hysteresis curves of Fe_3_O_4_ and Fe_3_O_4_@PDA, and the remanence and coercivity were close to zero, indicating that the materials exhibit a superparamagnetic character [33]. As can be observed in the lower right side of Fig. 3, Fe_3_O_4_@PDA was dispersed in water, affording a homogeneous suspension. Although the saturated magnetization value of Fe_3_O_4_@PDA was less than that of Fe_3_O_4_, Fe_3_O_4_@PDA was separated from wastewater by using an external magnetic field in 3 min. After removing the applied magnetic field, Fe_3_O_4_@PDA was rapidly dispersed into the solution, facilitating the collection, regeneration, and reuse of the adsorbents.

### Adsorption of Cd^2+^ in Batch Systems

The adsorption capacity of the adsorbents for heavy metal ions is mainly affected by factors such as reaction time, heavy metal-ion concentration, adsorbent dose, pH, and reaction temperature. Hence, batch adsorption experiments are carried out on the adsorbent to examine the effect of the above factors on the reaction, as well as the kinetics, isotherm, and thermodynamic properties of the adsorbent.

### Effect of Contact Time and Kinetics Study

**Fig. 4:**
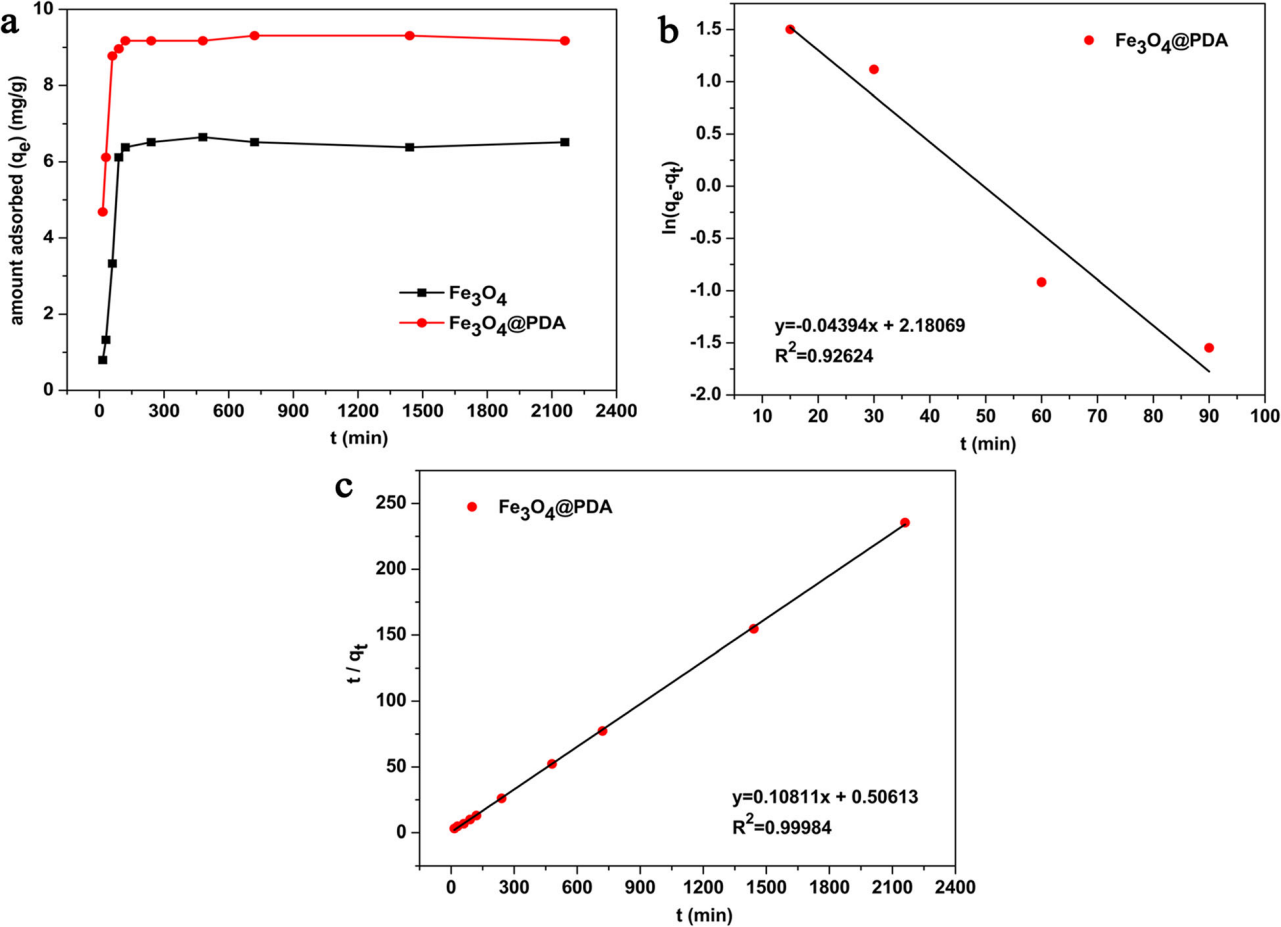
**a** Effect of the reaction time on the adsorption capacity of adsorbents (Fe_3_O_4_ and Fe_3_O_4_@PDA), **b** pseudo-first-order kinetics, and **c** pseudo-second-order kinetics of Fe_3_O_4_@PDA

Adsorption time is one of the important factors that affect the performance of an adsorbent. Figure 4a shows the effect of the adsorption time on adsorption capacity. From this figure, the adsorption capacity of Fe_3_O_4_ and Fe_3_O_4_@PDA for Cd^2+^ increased with time and tended to level after a certain time. Both adsorbents exhibited a more rapid adsorption rate before 90 min, and then the adsorption rates tended to be flat. Fe_3_O_4_ and Fe_3_O_4_@PDA reached the adsorption equilibrium at 240 and 120 min, respectively, because the difference in the concentration between Cd^2+^ and the solution on the adsorbent surface at the start of adsorption leads to the rapid movement of cadmium from the solution to the adsorbent surface. At the same time, a large number of adsorption active sites are present on the adsorbent surface, and Cd^2+^ in the aqueous solution exhibits a greater chance of occupying these sites. With the increase in the adsorption time, the active sites were predominantly occupied, and the positive charge on the adsorbed metal ions also repelled the metal ions in the water, increasing the difficulty in the adsorption of Cd^2+^ by the adsorbents. Hence, the adsorption rate gradually decreases and eventually reaches equilibrium. As Fe_3_O_4_@PDA comprises polar groups such as amino and hydroxyl groups, the affinity for water is greater than that for Fe_3_O_4_, which is beneficial for the contact of cadmium ions on the adsorbent surface; therefore, the equilibrium time of Fe_3_O_4_@PDA is shorter.

To analyze the adsorption kinetics of Fe_3_O_4_@PDA, adsorption data were simulated by the pseudo-first-order and pseudo-second-order kinetics models, which were expressed in Eqs. (2) and (3), respectively [51, 52].

2 $$ \ln \left({q}_e-{q}_t\right)=\ln {q}_{\mathrm{e}}-{k}_1t $$

3 $$ \frac{t}{q_t}=\frac{1}{k_2{q}_e^2}+\frac{t}{q_e} $$

where *k*_1_ (min^−1^) is the pseudo-first-order rate constant, *k*_2_ (g·mg^−1^·min^−1^) is the pseudo-second-order rate constant, and *q*_*e*_ and *q*_*t*_ represent the loading of Cd^2+^ at equilibrium and at time *t*, respectively.

A linear relationship between ln(*q*_*e*_ − *q*_*t*_) and *t* at different initial cadmium concentrations was observed (Fig. 4b). Figure 4c shows a graph obtained by the further analysis of *q*_*t*_ versus *t* using the pseudo-second-order rate law. The parameter values for the pseudo-first-order and pseudo-second-order kinetics were determined by the slope and intercept of the corresponding curve, respectively. Table 1 summarizes the results obtained. The correlation coefficient for the pseudo-second-order kinetics model (*R*^2^ = 0.9998) was greater than that of the pseudo-first-order kinetics model (*R*^2^ = 0.9262). Compared with *q*_*e*_^cal^ (8.852 mg/g) observed for the pseudo-first-order kinetics, *q*_*e*_^cal^ (9.250 mg/g) for the pseudo-second-order kinetics model was similar to the experimental value of *q*_*e*_ (9.176 mg/g). Therefore, the adsorption kinetics of Fe_3_O_4_@PDA follows the pseudo-second-order model, indicating that the adsorption of Cd^2+^ by Fe_3_O_4_@PDA is possibly consistent with chemical adsorption; that is, electrons are shared or exchanged between the adsorbent and adsorbate, and Cd^2+^ is adsorbed by the formation of covalent bonds or ion exchange [53].

**Table 1:** Kinetics adsorption parameters of Cd^2+^ by Fe_3_O_4_@PDA

*q*_*e*_^exp^ (mg/g)	Pseudo-first-order			Pseudo-second-order		
	*K*_1_ (min^−1^)	*q*_*e*_^cal^ (mg/g)	*R*^2^	*K*_2_ (g·mg^−1^·min^−1^)	*q*_*e*_^cal^ (mg/g)	*R*^2^
9.176	0.04394	8.852	0.9262	0.02309	9.250	0.9998

### Effect of Concentration and Adsorption Isotherms

**Fig. 5:**
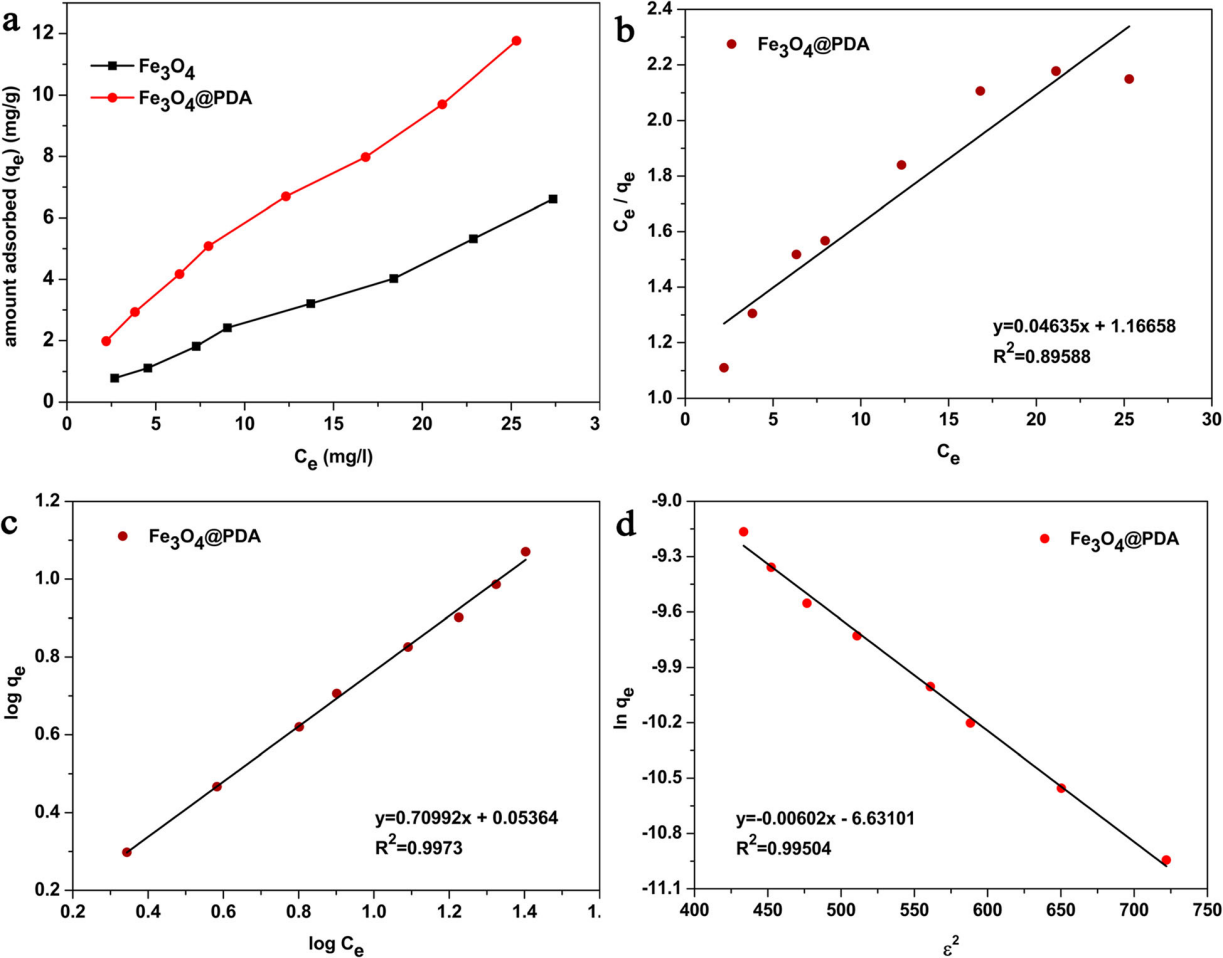
**a** Effect of the Cd^2+^ concentration on the adsorption capacity, **b** Langmuir isotherm, **c** Freundlich isotherm, and **d** D-R isotherm for the adsorption of Cd^2+^ by Fe_3_O_4_@PDA

The adsorption performance of the adsorbents (i.e., Fe_3_O_4_ and Fe_3_O_4_@PDA) was determined at Cd^2+^ concentrations ranging from 3 to 30 mg/L. Figure 5a shows the experimental result obtained. With the increase in the Cd^2+^ concentration, the *q*_*e*_ of the adsorbents increased because increased amounts of Cd^2+^ were available at high concentrations, increasing the possibility of contact between Cd^2+^ and the adsorbent active sites, and high concentration of cadmium also provided a higher driving force for the ions from the solution to the adsorbent surface. In addition, the adsorption capacity of Fe_3_O_4_@PDA was greater than that of Fe_3_O_4_ under the same adsorption conditions, indicating that the surface-dopamine-modified magnetic nanoparticles are more favorable for removing cadmium ions.

To understand the mechanism via which cadmium was adsorbed on Fe_3_O_4_@PDA, three isotherm adsorption models (i.e., Langmuir, Freundlich, and Dubinin–Radushkevich, respectively) were employed in this study to describe the adsorption equilibrium of Fe_3_O_4_@PDA. These isotherm equations were expressed as follows:

4 $$ \frac{C_e}{q_e}=\frac{1}{bq_m}+\frac{C_e}{q_m} $$

5 $$ \log\ {q}_e=\log k+\frac{1}{n}\log {C}_e $$

6 $$ \ln\ {q}_e=\ln {q}_d-{K}_d{\varepsilon}^2 $$

where *q*_*e*_ (mg/g) and *C*_*e*_ (mg/L) are the adsorption capacity and Cd^2+^ concentration at equilibrium, respectively; *b* (L/mg) is the Langmuir adsorption equilibrium constant; and *q*_*m*_ (mg/g) is the maximum adsorption capacity. The *K* [(mol/g)/(mol/L)^1/n^] value can be regarded as the adsorption amount with Cd^2+^ as the unit concentration and 1/*n* as the indicator of the adsorption strength. *q*_*d*_ (mol/g) is the maximum adsorption capacity, *K*_*d*_ (mol^2^/kJ^2^) is a constant related to adsorption energy, and *ε* is the Polanyi potential (*ε* = *RT* ln(1 + 1/*C*_*e*_), where the unit of *C*_*e*_ is mol/L).

In addition, the equilibrium parameter *R*_*L*_ [1/(1 + *bC*_*o*_)], highest initial solute concentration in the concentration gradient *C*_*0*_ (mg/L)), and the mean free energy of adsorption, *E*_*d*_ [(2*K*_*d*_)^−1/2^, kJ/mol] were estimated. *R*_*L*_ was utilized to determine whether the adsorption process is favorable. *E*_*d*_ can be used to determine the type of adsorption. *E*_*d*_ values of 1–8 kJ/mol were indicative of physical adsorption (such as van der Waals forces), while those of 8–16 kJ/mol were indicative of ion exchange. An *E*_*d*_ value of between 20 and 40 kJ/mol revealed that the adsorption reaction is chemisorption [54].

The isothermal curves constructed with the Langmuir, Freundlich, and D-R models correspond to b, c, and d in Fig. 5, respectively. Table 2 summarizes the calculated isothermal parameters. The correlation coefficient from the fitting of the curve revealed that the Freundlich model (*R*^2^ = 0.9973) is more suitable than the Langmuir model (*R*^2^ = 0.8959) and D-R model (*R*^2^ = 0.9950) for describing the adsorption of Cd^2+^ by Fe_3_O_4_@PDA. Hence, the adsorption of Cd^2+^ can be described by the Freundlich isotherm, indicating that Cd^2+^ is adsorbed on an uneven surface by the multilayer and that the energy distribution for the surface-active sites of the adsorbent is uneven [55]. Simultaneously, *n* > 1 reflects the high affinity between the adsorbate and adsorbent; hence, adsorption is favorable [56, 57]. The maximum adsorption capacity (*q*_*m*_) obtained by the Langmuir model was 21.58 mg/g. The *R*_*L*_ value was between 0 and 1, indicative of the advantageous reaction of Fe_3_O_4_@PDA to adsorb Cd^2+^ [58]. In addition, in this study, the *E*_*d*_ value was calculated to be 9.114 kJ/mol, which was within the range of values reflecting the ion exchange mechanism (8–16 kJ/mol) and was similar to that reflecting the electrostatic adsorption (1–8 kJ/mol). Hence, by the combination of the *E*_*d*_ value and the preceding analysis for pseudo-second-order kinetics, the adsorption of Cd^2+^ on Fe_3_O_4_@PDA is predominated by ion exchange and electrostatic adsorption mechanisms.

**Table 2:** The Langmuir, Freundlich, and D-R isotherm parameters for the adsorption of Cd^2+^ by Fe_3_O_4_@PDA

Langmuir model				Freundlich model			D-R model			
*b* (L/mg)	*q*_*m*_ (mg/g)	*R*_*L*_	*R*^2^	*K* (mg/g)	*n*	*R*^2^	*q*_*d*_ (mol/g)	*K*_*d*_ (mol^2^/kJ^2^)	*E*_*d*_ (kJ/mol)	*R*^2^
0.03973	21.58	0.4562	0.8959	1.132	1.409	0.9973	1.319 × 10^–3^	6.02 × 10^–3^	9.114	0.9950

The *q*_*m*_ value of Fe_3_O_4_@PDA was compared with the adsorption capacity of Cd^2+^ for other previously reported adsorbents (Table 3). The *q*_*m*_ value widely varied for different adsorbents. Generally, commercial exchange resins and activated carbons exhibit higher adsorption capacities due to their higher specific surface areas, but the recovery and separation of these resins and carbons are not possible. The *q*_*m*_ value of Fe_3_O_4_@PDA adsorbing Cd^2+^ was relatively reasonable (Table 3), and Fe_3_O_4_@PDA could be recovered by a magnetic field, indicating that the adsorbent can be possibly applied for the removal of Cd^2+^ from wastewater.

**Table 3:** Comparison of the maximum adsorption capacities of Fe_3_O_4_@PDA with some adsorbents cited in the literature

Adsorbents		*q*_*m*_ (mg/g)	pH	*C*_*0* (Cd)_ (mg/L)	References
Nano-hydroxyapatite nanorods		92	5.8	100–500	[1]
Nano-hydroxyapatite chitosan composites		122			
Fe_3_O_4_@SiO_2_@m-SiO_2_–NH_2_		884.906	6	20–843	[20]
Fly ash/chitosan (A-FA/Ch) composite		87.72	8	25–600	[21]
Surface-modified Eucalyptus seeds by sulfuric acid (SMES-S)		71.15	5	20–100	[52]
Surface-modified Eucalyptus seeds by hydrochloric acid (SMES-H)		64.16			
Nano zero-valent iron particles		769.2	–	25–450	[57]
Biopolymeric sorbent: sporopollenin		2.23	7	0–250	[59]
Poly (sodium acrylate)–graphene oxide		225.7	6	20–600	[60]
Zero-valent iron-coated biochars (magnetic ones)	MBC_1_	38.00	5	50–600	[61]
	MBC_2_	41.25			
Multiwalled carbon nanotubes (MWCNTs)	Raw-MWCNT	1.260 ± 0.02	8	0.1–5	[62]
	O-MWCNT	22.39 ± 0.36			
	E-MWCNT	21.67 ± 0.40			
Sawdust of *Pinus sylvestris*		15.27	4	1–50	[63]
		19.08	5		
		6.72	7		
Mustard husk		42.85	6	1–5	[64]
Mercaptoacetic-acid-modified orange peel (MOP)		136.05	7	50–1000	[65]
Polydopamine-modified Fe_3_O_4_		21.58	7	3–30	This study

### Effect of Temperature and Thermodynamic Parameters

**Fig. 6:**
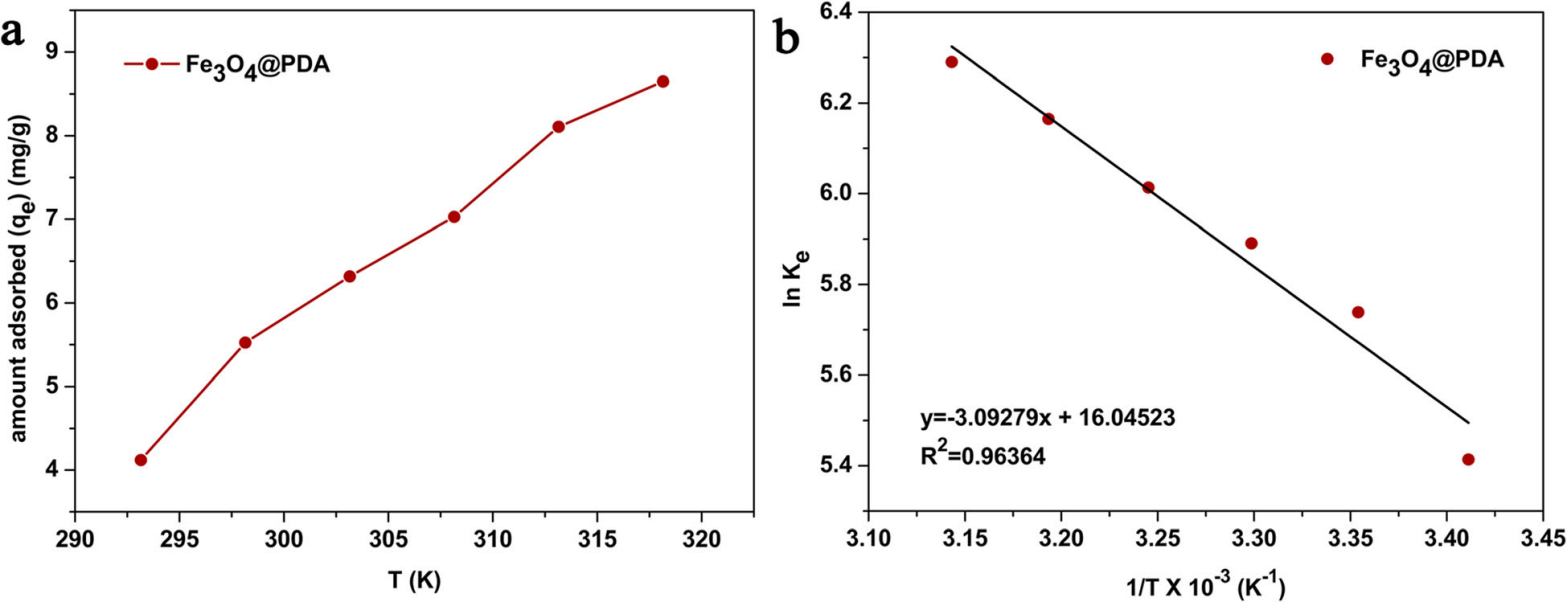
**a** Effect of temperature on the adsorption of Cd^2+^ and **b** relationship between ln*K*_*e*_ and 1/*T* for the Cd^2+^ adsorption on Fe_3_O_4_@PDA

Temperature is crucial for adsorption. Therefore, the effect of temperatures in the range of 293.15–318.15 K on the adsorption performance of Fe_3_O_4_@PDA is examined. With increasing temperature, the adsorption capacity of the adsorbents for Cd^2+^ increased (Fig. 6a). This trend indicated that the adsorption of Cd^2+^ by Fe_3_O_4_@PDA is an endothermic process, and high temperature is favorable for the adsorption of Cd^2+^. In addition, high temperature promotes the mobility of metal ions, thereby increasing the possibility of contact between Cd^2+^ and active sites as well as the adsorption capacity of the adsorbents.

At the same time, various thermodynamic parameters were calculated to examine the thermodynamics properties of Fe_3_O_4_@PDA, such as entropy (*∆S°*), enthalpy (*∆H°*), and Gibbs free energy (*∆G°*). The calculated equations were expressed as follows:

7 $$ {K}_e=\frac{q_e}{c_e} $$

8 $$ \Delta {G}^o=- RT\ln {K}_e $$

9 $$ \Delta {G}^o=\Delta {H}^o-T\Delta {S}^o $$

The van ’t Hoff equation can be derived from Eqs. (8) and (9) as follows:

10 $$ \ln {K}_e=-\frac{\Delta {H}^o}{RT}+\frac{\Delta {S}^o}{R} $$

where *q*_*e*_ (mg/g) is the adsorption capacity of the adsorbent for cadmium ions at the reaction equilibrium, *C*_*e*_ (mg/L) is the concentration of cadmium ions in the solution at equilibrium, *K*_*e*_ (mL/g) is the thermodynamic equilibrium constant (the value depends on the temperature), *T* (K) is the absolute temperature, and *R* [8.314 J/(mol·K)] is the gas constant.

Figure 6b shows the relationship between ln*K*_*e*_ and 1/*T* of Cd^2+^ adsorbed by Fe_3_O_4_@PDA. After fitting, the straight line with a linear correlation coefficient *R*^2^ of 0.9636 was obtained. The *R*^2^ value revealed that ln*K*_*e*_ exhibits a basic linear relationship with 1/*T*. The thermodynamic parameters were calculated from the slope and intercept of the curve. Table 4 summarizes the results obtained. With increasing temperature, the *∆G°* of cadmium ions decreased from − 13.196 to − 16.638 kJ/mol (Table 4), indicating that the adsorption of Cd^2+^ by Fe_3_O_4_@PDA is a spontaneous reaction and that the degree of a spontaneous reaction increases with temperature. The increase of *K*_*e*_ with temperature and *ΔH°* > 0 revealed that the adsorption is an endothermic reaction as Cd^2+^ is solvated in water to form hydrated ions. For the adsorption of the ions, dehydration is required to some extent. Hence, it is crucial to absorb heat, which is provided by temperature. The higher the temperature, the higher the degree of dehydration for hydrated ions, which is also one of the reasons for the favorable adsorption at high temperatures [59]. The *ΔH°* value can be used to determine whether adsorption is physical or chemical adsorption. The *ΔH°* value for physical adsorption is between 2 and 21 kJ/mol, while the *ΔH*° value for chemisorption is between 80 and 200 kJ/mol [66]. The *ΔH°* value calculated in this experiment was ~ 25.714 kJ/mol, suggesting that the type of adsorption is physico-chemical adsorption rather than pure physical or chemical adsorption. The entropy change *∆S°* was positive [0.1334 kJ/(K·mol)], indicating that adsorption increases the chaos on the adsorbent surface and that the randomness for the adsorption of cadmium ions at the solid–solution interface increases at the active sites of the Fe_3_O_4_@PDA [67]. At the same time, the positive value of *∆S°* also revealed that ion exchange occurs on the adsorbent surface [56].

**Table 4:** Thermodynamic parameters for the adsorption of Cd^2+^ by Fe_3_O_4_@PDA

*T* (K)	*K*_*e*_ (mL/g)	*∆G°* (kJ/mol)	*∆H°* (kJ/mol)	*∆S°* [kJ/(K·mol)]
293.15	224.55	− 13.196	25.714	0.13340
298.15	310.56	− 14.224		
303.15	361.64	− 14.847		
308.15	408.81	− 15.406		
313.15	475.81	− 16.051		
318.15	539.22	− 16.638		

### Effect of Adsorbent Dose

**Fig. 7:**
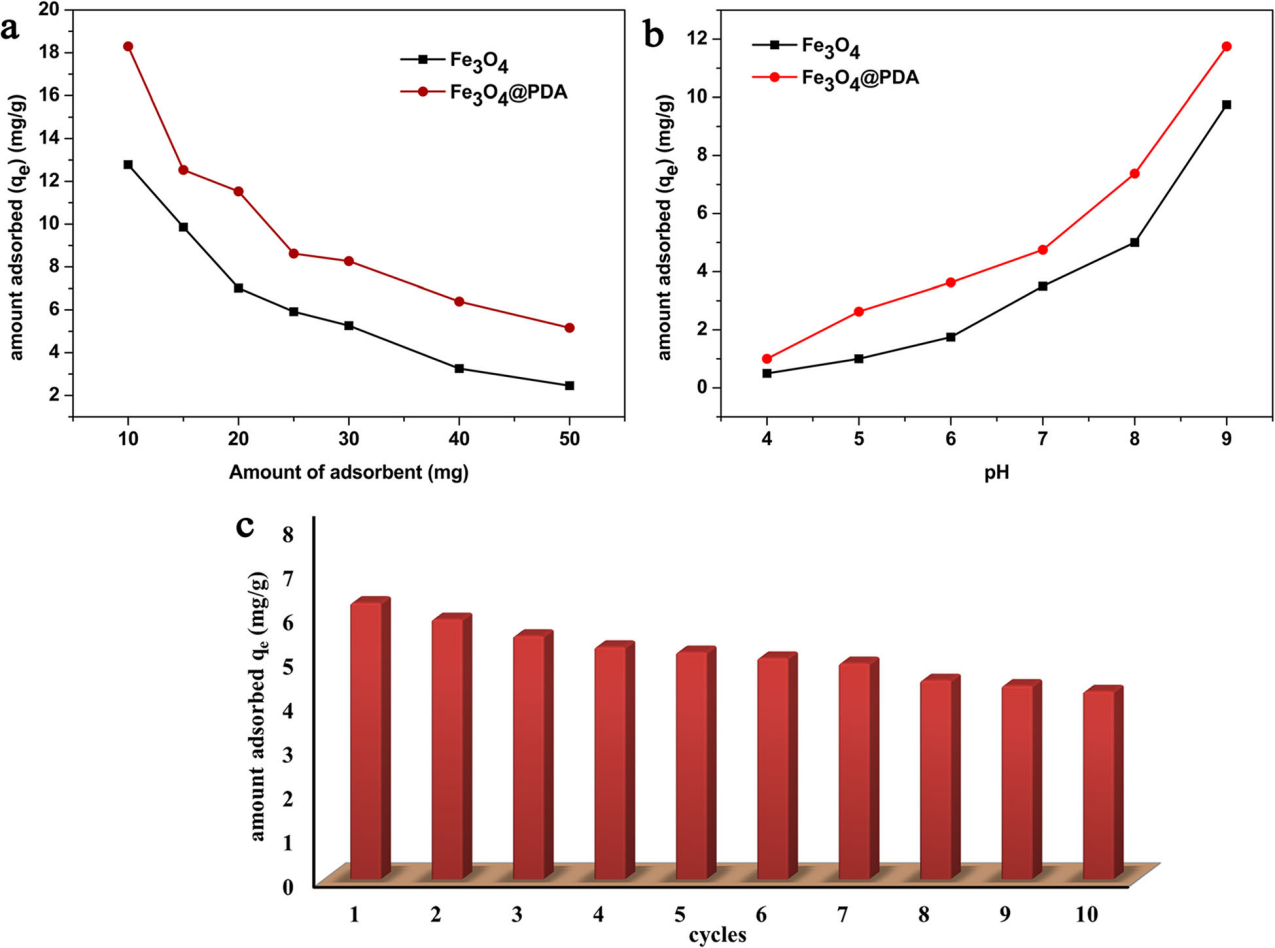
**a** Effect of different adsorbent doses on the adsorption of Cd^2+^, **b** effect of pH on the uptake of metal ions by the adsorbents, and **c** variation of the adsorption capacity of Fe_3_O_4_@PDA for Cd^2+^ with the number of adsorption–desorption cycles

Figure 7a shows the comparison of the effects of different adsorbent doses (10–50 mg) on the adsorption of cadmium ions. The course of the curve shown in Fig. 7a indicated that the adsorption capacity gradually decreases with the increase in the adsorbent dose. The increased adsorbent dose provided additional active sites, but at high adsorbent concentrations, the adsorbent underwent agglomeration, thereby decreasing the unoccupied adsorption active sites and effective surface area. In addition, if the initial concentration and volume of Cd^2+^ are constant, the number of cadmium ions contacted and adsorbed by the adsorbent per unit mass decreases with the increase in the adsorbent dose, and the active sites of the adsorbents are not saturated. Hence, with the increase in the adsorbent dose, the adsorption capacity of Cd^2+^ gradually decreases. Under the same dose of adsorbents, the adsorption capacity of Fe_3_O_4_@PDA for cadmium ions was greater than that of Fe_3_O_4_, indicating that Fe_3_O_4_@PDA exhibits advantages over bare Fe_3_O_4_ in cadmium removal.

### Effect of pH

The solution pH is also one of the most important factors affecting adsorption. The adsorption of Cd^2+^ is mainly affected by the surface charges on the adsorbents, and the surface charges of the adsorbents are affected by the solution pH. Considering the degree of tolerance for adsorbents to acid and base, the chemical states of cadmium ions in an aqueous solution [i.e., Cd^2+^, Cd (OH)^+^, Cd (OH)_2_, and Cd (OH)_3_^−^] [60], and the actual conditions of environmental water samples, pH values of between 4 and 9 were selected to investigate the adsorption of Cd^2+^ by the adsorbents.

Figure 7b shows the experimental results. With the increase in the solution pH, the adsorption amount of the adsorbents on Cd^2+^ significantly increased. At low solution pH, the concentration and activity of H^+^ in the solution were extremely high, which can compete with Cd^2+^ for adsorption and occupy the active site on the adsorbent surface, leading to the low adsorption capacity of Cd^2+^ by the adsorbent [61]. At low solution pH, the adsorption of cadmium must overcome the repulsive force between the positively charged Fe_3_O_4_@PDA surface and Cd^2+^ through chemical interactions with sufficient energy rather than through electrostatic attractions [68]. At solution pH values of between 6.0 and 8.0, the main chemical states of cadmium were Cd^2+^ (minor) and Cd (OH)^+^ (major) [69]. As the affinity of Cd (OH)^+^ was better than that of Cd^2+^, it can be adsorbed on the adsorbent surface not only by electrostatic adsorption and ion exchange but also by hydrogen bonds. With the further increase in the solution pH, the protonation sites on the adsorbent surface decreased, and the negative charge increased, facilitating the adsorption of Cd^2+^ and Cd (OH)^+^ on the deprotonation active sites of the adsorbent by electrostatic adsorption [70]. Hence, the increase in pH is beneficial to the adsorption of heavy metal cadmium. The precipitation of cadmium at pH 8.8 was calculated from the precipitation constant of Cd (OH)_2_(s) (*K*_sp_ = 7.2 × 10^−15^) and the initial Cd^2+^ concentration (20 mg/L). Thus, at a solution pH from 8.0 to 9.0, the adsorption capacities of both adsorbents sharply increase (Fig. 7b). Moreover, the high adsorption amount at pH 9.0 resulted from the formation of a Cd (OH)_2_ precipitate rather than the adsorption of cadmium on the adsorbent. Nevertheless, at a solution pH between 4.0 and 8.8, the adsorption capacity of Fe_3_O_4_@PDA was greater than that of bare Fe_3_O_4_, indicating that the Fe_3_O_4_@PDA adsorbent can be used in the wide pH range of 4.0–8.8 for treating Cd^2+^-containing wastewater.

Combined with the kinetics, isotherm model, and thermodynamic analysis, the adsorption of cadmium by Fe_3_O_4_@PDA is a physico-chemical process, and the adsorption mechanism is mainly electrostatic adsorption and ion exchange, supplemented by complexation. At the same time, combined with the effect of pH on the adsorption result, the adsorption mechanism can be obtained from Eqs. 11–18 [62–64]:

11 $$ -{NH}_2+{H}^{+}\to -{NH}_3^{+} $$

12 $$ -{NH}_2+{Cd}^{2+}\to -{NH}_2{Cd}^{2+} $$

13 $$ -{NH}_2+{OH}^{-}\to -{NH}_2{OH}^{-} $$

14 $$ -{NH}_2{OH}^{-}+{Cd}^{2+}\to -{NH}_2{OH}^{-}{Cd}^{2+} $$

15 $$ -{NH}_2{OH}^{-}+{CdOH}^{+}\to -{NH}_2{OH}^{-}{CdOH}^{+} $$

16
17
18

To verify the occurrence of ion exchange, the pH of the residual solutions at pH 6, 7, and 8 was measured after the completion of the experiment. The result revealed that the solution pH slightly decreases after adsorption, confirming the presence of the proposed mechanism for (17) and (18). This result indicated that the reactions of (17) and (18) considerably contribute to the overall adsorption process.

### Reusability and Stability Studies

The reusability and stability of adsorbents are crucial for the industrial treatment of heavy metal wastewater. The inhibition of the heavy metal adsorption on Fe_3_O_4_@PDA at low pH indicated that acid treatment is a viable method for regenerating heavy metal-loaded adsorbents. Hence, in this experiment, 0.5 M HCl is used as the desorbent, and 10 adsorption–desorption cycles are carried out using the Fe_3_O_4_@PDA adsorbent. With the increase in the number of experiments, the adsorption capacity gradually decreased (Fig. 7c), possibly related to the incomplete desorption of cadmium ions adsorbed on the adsorbent surface. After the completion of the 10th cycle, the adsorption capacity of the adsorbents was reduced from 6.25 to 4.25 mg/g, and the adsorption rate only decreased by 3.6% compared with that of the initial cycle, indicating that Fe_3_O_4_@PDA exhibits good reusability and provides a basis for the practical applications of Fe_3_O_4_@PDA.

TEM images of the adsorbent were recorded after 10 adsorption–desorption cycles (Fig. 1e). After 10 desorption cycles of Fe_3_O_4_@PDA in an acidic environment of 0.5 M HCl, the PDA layer was preserved, but the Fe_3_O_4_ core was corroded. Although the Fe_3_O_4_@PDA core structure was damaged, the core–shell structure was complete, and the adsorption efficiency did not change considerably, indicating that PDA exhibits a good protective effect on the Fe_3_O_4_ core and that Fe_3_O_4_@PDA can stably exist in an acidic environment. The results showed that Fe_3_O_4_@PDA exhibits excellent stability and adsorption properties, providing the basis for the practical applications of Fe_3_O_4_@PDA because if the adsorbent exhibits good reusability and stability, it will effectively decrease the cost of industrial applications.

## Conclusions

In conclusion, a highly stable, hydrophilic functionalized magnetic nano-adsorbent (Fe_3_O_4_@PDA) was synthesized by a simple, safe, and environmentally friendly method in this study. Results revealed that the Cd^2+^ adsorption is dependent on the contact time, initial Cd^2+^ concentration, temperature, adsorbent dose, and solution pH. The adsorption performance of Cd^2+^ on Fe_3_O_4_@PDA was better than that of bare Fe_3_O_4_, which was related to the presence of active sites such as phenolic hydroxyl groups (electron negative groups) and amino groups on the Fe_3_O_4_@PDA surface. In kinetics studies, adsorption equilibrium was achieved at 120 min, and the adsorption capacity of Cd^2+^ onto Fe_3_O_4_@PDA reached up to 9.176 mg/g. The adsorption of Cd^2+^ followed the pseudo-second-order kinetics model. The adsorption of Cd^2+^ onto Fe_3_O_4_@PDA was consistent with the Freundlich isotherm, and the maximum adsorption capacity obtained by the Langmuir model was 21.58 mg/g. Thermodynamic analyses indicated that the reaction is spontaneous and endothermic. Meanwhile, the possible adsorption mechanism was also proposed on the basis of the kinetics, D-R isotherm model, and thermodynamic results, i.e., Cd^2+^ was adsorbed on the Fe_3_O_4_@PDA surface-active site by electrostatic adsorption, ion exchange, and chelation. Furthermore, 10 adsorption–desorption cycles were carried out using the Fe_3_O_4_@PDA nano-adsorbent for water samples containing cadmium. The adsorption rate of the adsorbent was only decreased by 3.6%, indicating that Fe_3_O_4_@PDA exhibits good adsorption stability and reusability, thereby reducing costs. The dissolution of Fe_3_O_4_@PDA in 0.5 M HCl indicated that the adsorbent can be treated harmlessly, avoiding secondary environmental pollution. The results revealed that Fe_3_O_4_@PDA exhibit immense potential for the treatment of cadmium-containing wastewater.

## Data Availability

All data supporting the conclusions of this article are included within the article.

## Abbreviations

BET: Brunauer–Emmet–Teller measurements
Fe_3_O_4_: FeO·Fe_2_O_3_
Fe_3_O_4_@PDA: Fe_3_O_4_ modified with polydopamine
FTIR: Fourier transform infrared spectroscopy
TEM: Transmission electron microscopy
VSM: Vibrating sample magnetometer
XPS: X-ray photoelectron spectroscopy
XRD: X-ray diffraction
